# Supplementary material for: Enhancing Transcriptional Reprogramming of Mesenchymal Glioblastoma with Grainyhead-like 2 and HDAC Inhibitors Leads to Apoptosis and Cell-Cycle Dysregulation
Source: Genes (Basel). 2023 Sep 12;14(9):1787. doi: 10.3390/genes14091787 (PMC10530281; doi:10.3390/genes14091787)
Supplement: Supplementary file 1 [file genes-14-01787-s001.zip › genes-2573615-supplementary.pdf]

Fig S1

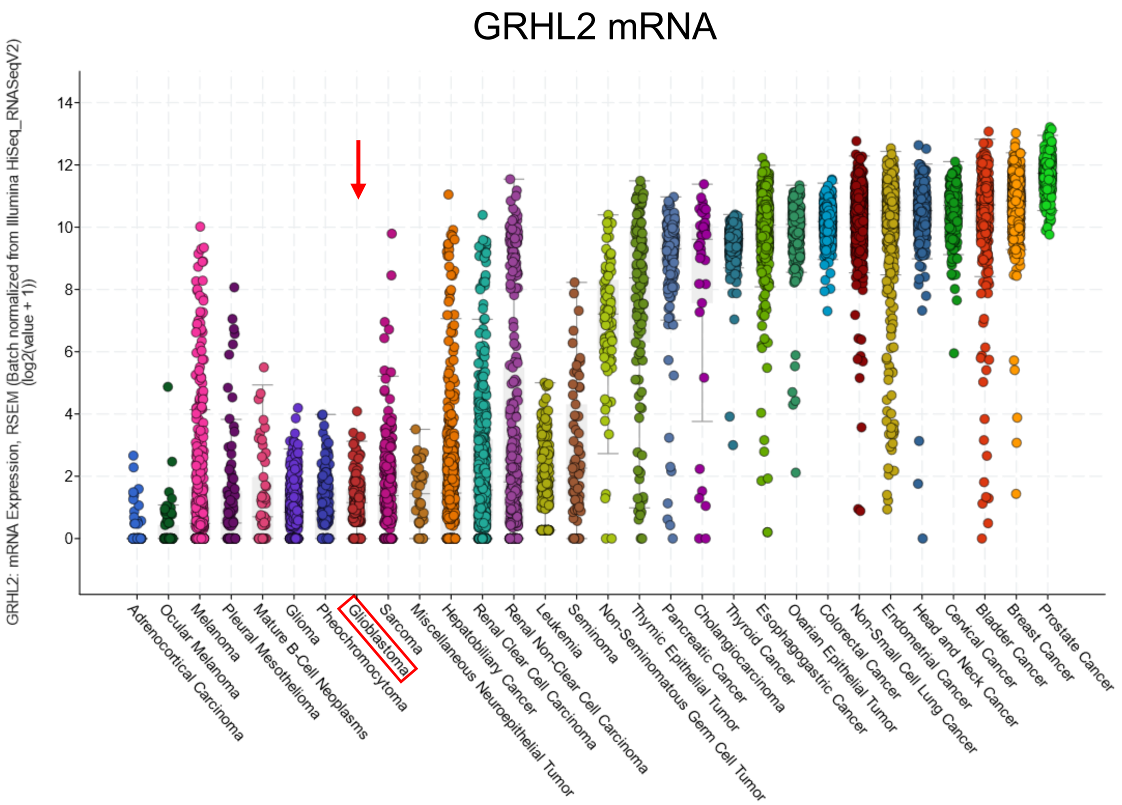

Figure S1. GRHL2 expression in patient tumors. Graph depicting GRHL2 mRNA expression in different cancers, color coded by tumor type. Data captured from TCGA cBioBortal. Cancer types are sorted according to median expression.

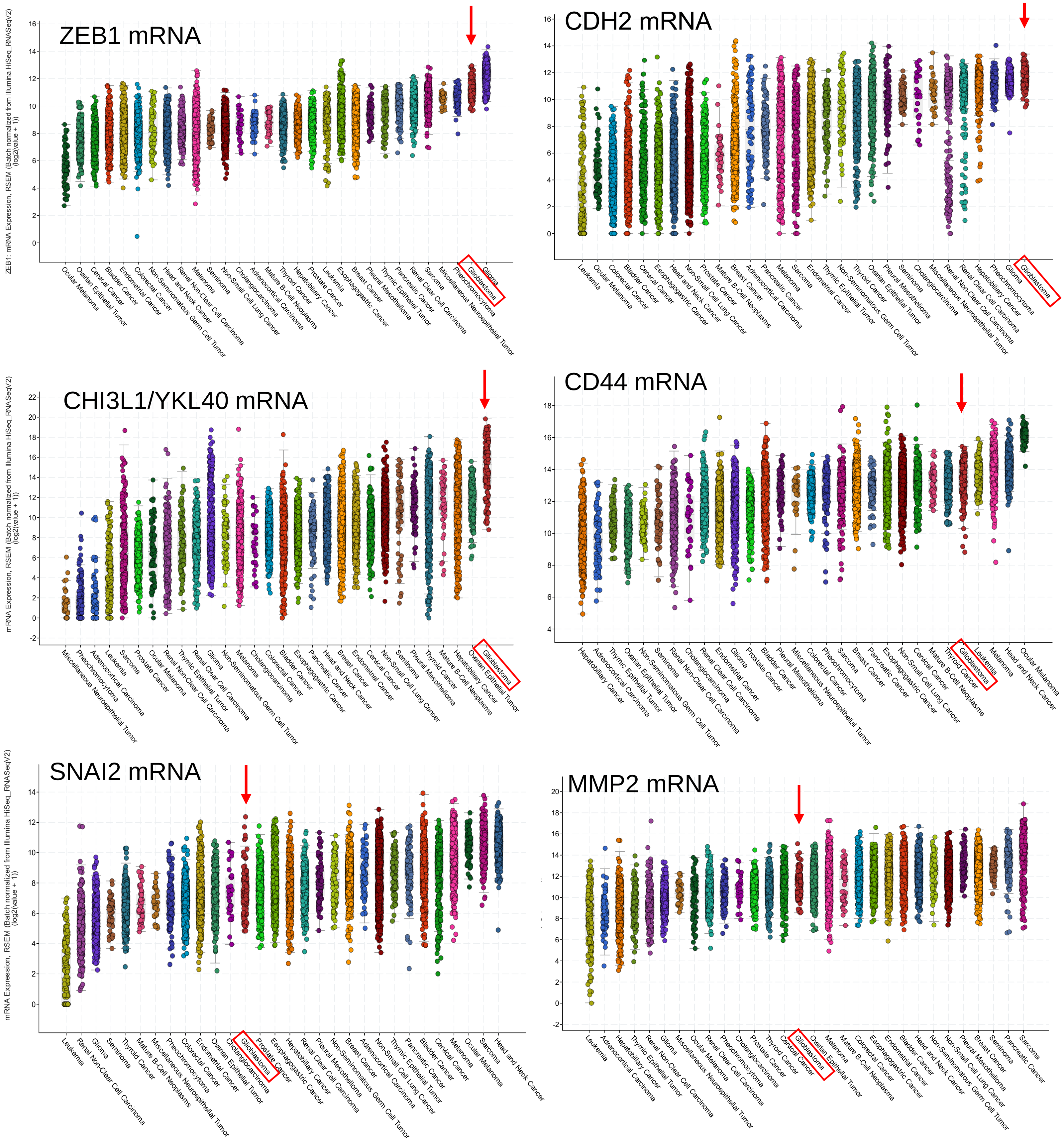

Figure S2. Select mesenchymal gene expression markers in patient tumors. mRNA expression in patient tumor types is color coded by tumor type. Data captured from TCGA cBioBortal. Cancer types are sorted according to median expression.

Fig S3

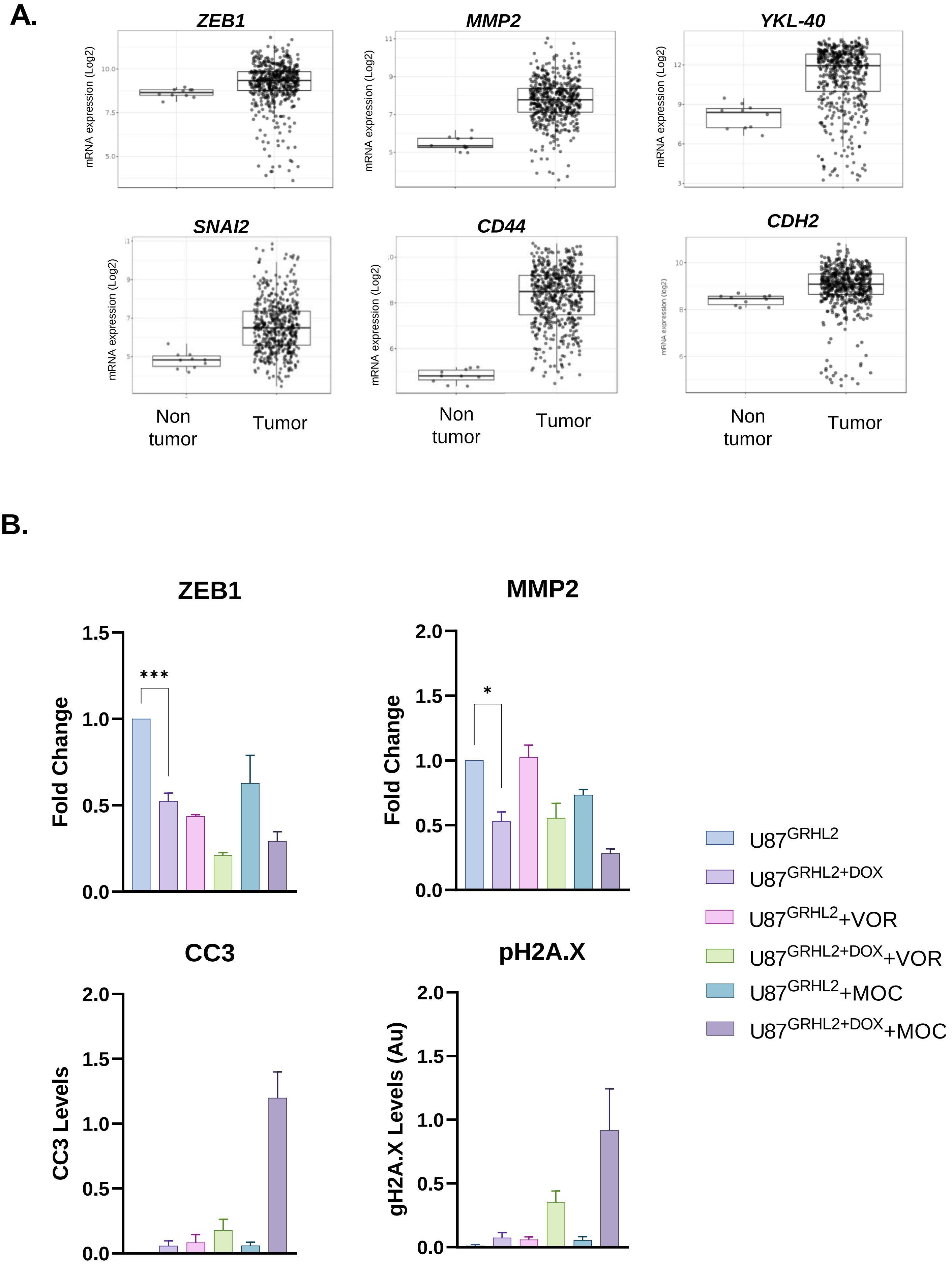

Figure S3. Proteins associated with the mesenchymal phenotype are elevated in patient GBM tumors but repressed by GRHL2 in U87 GBM cells. A. Graph depicting *CD44*, *MMP2*, *YKL-40*, *SNAI2*, *ZEB1* expression in non-tumor vs. GBM tissues. Data captured from GlioVis http://gliovis.bioinfo.cnio.es/ using the TCGA GBM dataset. **B.** Effect of GRHL2 expression in U87 cells on mesenchymal markers ZEB1 and MMP2, CC3 and pH2AX in U87 cells. Data are means ± SEM (n=3). * $p < 0.05$ t-test.

Fig S4

A.

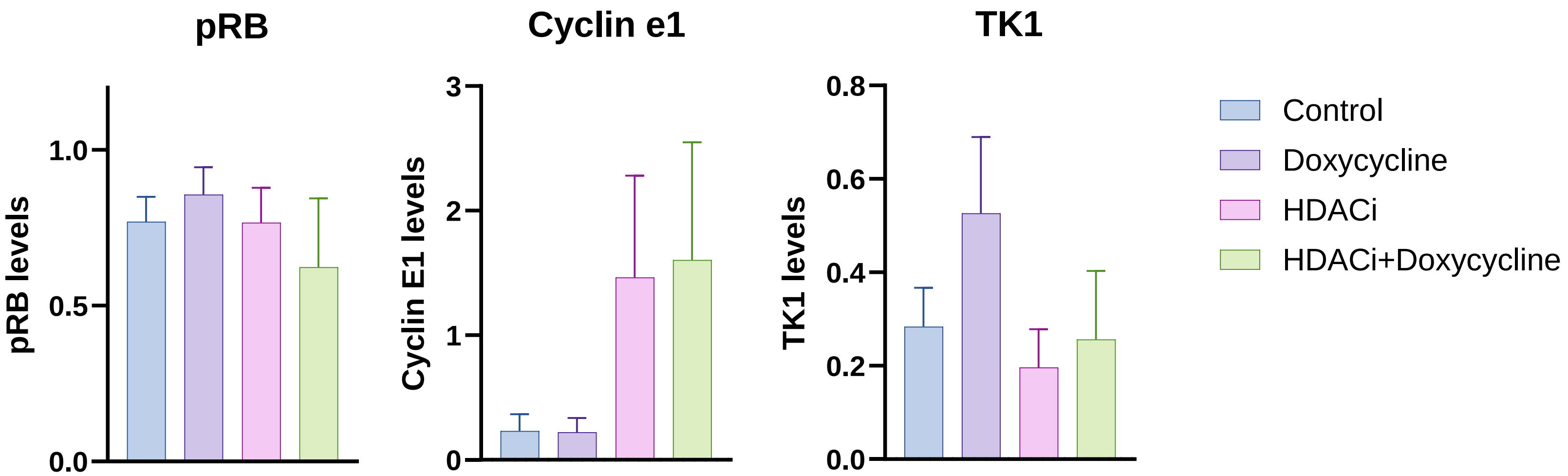

B.

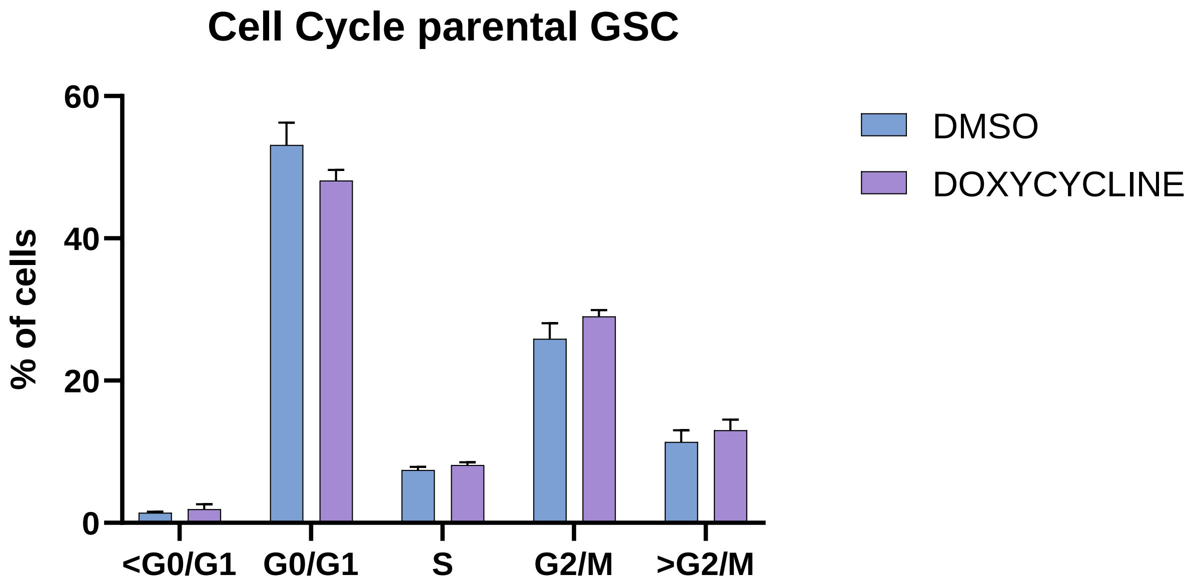

Figure S4. Doxycycline treatment of GSCs. A. Dox inducible GRHL2 expression in GSCs did not clearly affect cell cycle proteins pRB, cyclin e1, or TK1. **B.** Dox treatment of parental (not-inducible) GSCs does not significantly impact cell cycle parameters. GSCs were treated with doxycycline for 3 days followed by propidium iodide flow cytometry to assess cell cycle phase distribution.

Fig S5

Key Mitotic Proteins Identified	ID	Number	Not Induced Rep#1	Not Induced Rep#2	Not Induced Rep#3	GRHL2 Induced Rep#1	GRHL2 Induced Rep#2	GRHL2 Induced Rep#3
Nuclear mitotic apparatus protein 1	NUMA1	Q14980	0.0	0.0	0.0	7.9	2.6	6.4
Sister chromatid cohesion protein PDS5 homolog A	PDS5A	Q29RF7	1.2	0.0	1.4	3.9	2.6	3.2
Sister chromatid cohesion protein PDS5 homolog B	PDS5B	Q9NTI5	0.0	0.0	0.0	3.9	1.8	3.2
Mitotic checkpoint protein BUB3	BUB3	O43684	0.0	0.0	0.0	1.3	2.6	1.6
Dynamin-2	DYN2	P50570	0.0	0.0	0.0	0.7	0.9	1.6
DNA replication licensing factor MCM5	MCM5	P33992	3.6	1.7	4.1	8.5	6.2	7.2
DNA replication licensing factor MCM3	MCM3	P25205	4.8	0.0	1.4	9.9	2.6	5.6
DNA replication licensing factor MCM6	MCM6	Q14566	4.8	1.7	1.4	6.6	3.5	4.8
DNA replication licensing factor MCM7	MCM7	P33993	3.6	3.5	6.9	11.2	11.4	8.0
Structural maintenance of chromosomes protein 1A	SMC1A	Q14683	2.4	1.7	2.8	5.3	5.3	6.4
Importin subunit beta-1	IMB1	Q14974	2.4	1.7	1.4	5.3	4.4	4.8
Cytoplasmic dynein 1 heavy chain 1	DYHC1	Q14204	54.9	45.3	73.3	71.6	70.4	82.4

Figure S5. Key mitotic proteins identified by mass spectrometry from immunoprecipitates of inducible GRHL2. RIME immunoprecipitation of GRHL2 and mass spectrometry analysis from GRHL2-inducible cells treated with or without doxycycline for 72 h. Data are unique peptide counts. N = 3 independent replicates.

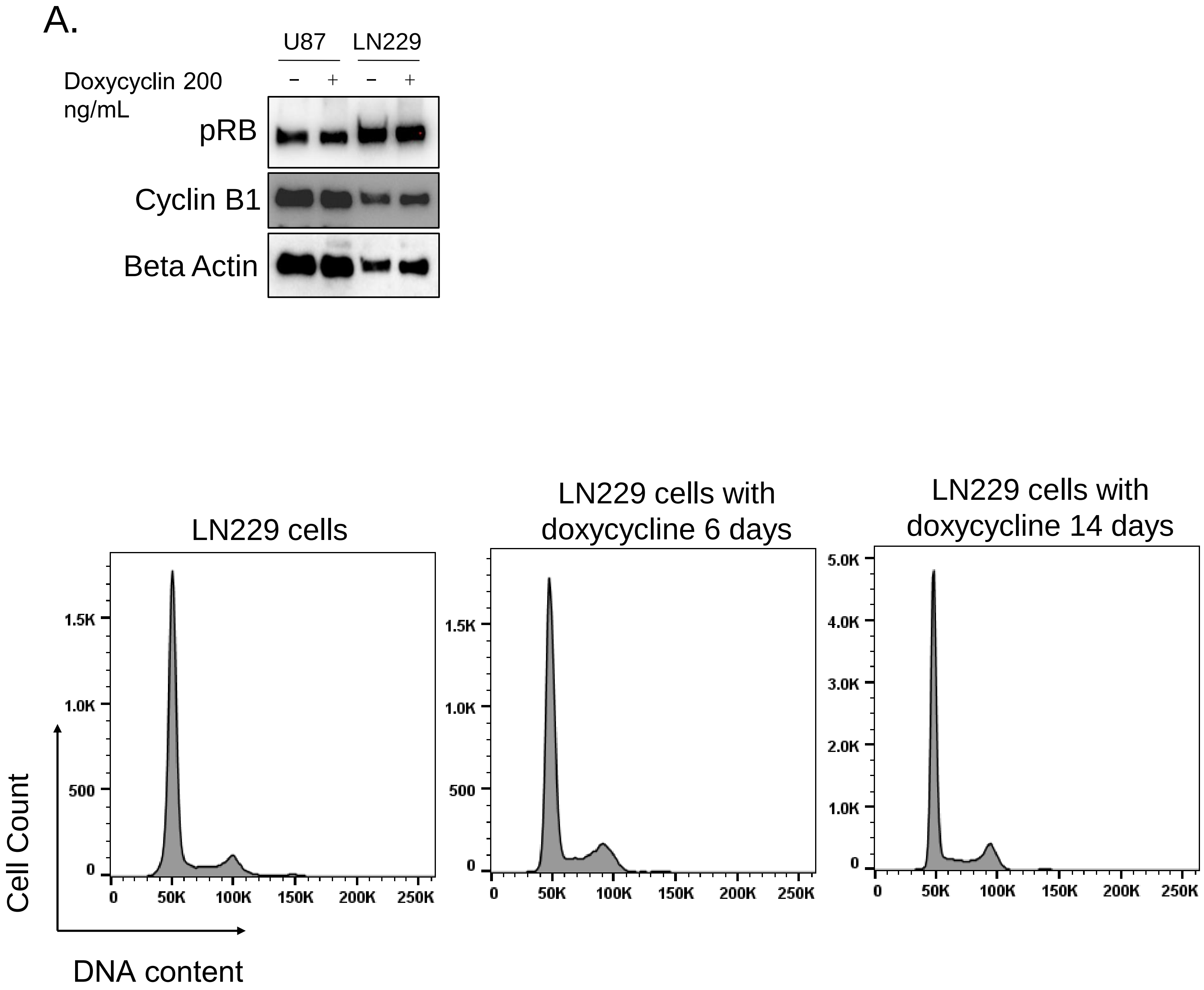

Figure S6. Effect of doxycycline on GBM cells. A. U87 and LN229 parental cells were treated with doxycyclin for 72 h followed by western blot for pRB and cyclin B1. B. LN229 cells were treated with doxycycline for the indicated time periods followed by cell cycle analysis with propidium iodide flow cytometry.
